# Numerical investigation of the tribological performance of micro-dimple textured surfaces under hydrodynamic lubrication

**DOI:** 10.3762/bjnano.8.232

**Published:** 2017-11-06

**Authors:** Kangmei Li, Dalei Jing, Jun Hu, Xiaohong Ding, Zhenqiang Yao

**Affiliations:** 1 School of Mechanical Engineering, Donghua University, 2999 North Renmin Road, Shanghai, 201620, China; 2 School of Mechanical Engineering, University of Shanghai for Science and Technology, 516 Jungong Road, Shanghai, 200093, China; 3 State Key Laboratory of Mechanical System and Vibration, Shanghai Jiao Tong University, 800 Dongchuan Road, Shanghai, 200240, China,; 4 School of Mechanical Engineering, Shanghai Jiao Tong University, 800 Dongchuan Road, Shanghai, 200240, China

**Keywords:** CFD simulation, hydrodynamic lubrication, micro-dimple array, surface texture, tribological performance

## Abstract

Surface texturing is an important approach for controlling the tribological behavior of friction pairs used in mechanical and biological engineering. In this study, by utilizing the method of three-dimensional computational fluid dynamics (CFD) simulation, the lubrication model of a friction pair with micro-dimple array was established based on the Navier–Stokes equations. The typical pressure distribution of the lubricant film was analyzed. It was found that a positive hydrodynamic pressure is generated in the convergent part of the micro-dimple, while a negative hydrodynamic pressure is generated in the divergent part. With suitable parameters, the total integration of the pressure is positive, which can increase the load-carrying capacity of a friction pair. The effects of the micro-dimple parameters as well as fluid properties on tribological performance were investigated. It was concluded that under the condition of hydrodynamic lubrication, the main mechanism for the improvement in the tribological performance is the combined effects of wedging and recirculation. Within the range of parameters investigated in this study, the optimum texture density is 13%, while the optimum aspect ratio varies with the Reynolds number. For a given Reynolds number, there exists a combination of texture density and aspect ratio at which the optimum tribological performance could be obtained. Conclusions from this study could be helpful for the design of texture parameters in mechanical friction components and even in artificial joints.

## Introduction

The wear caused by friction is considered to be the main reason for the failure of mechanical systems and the major source of energy loss [1]. Various methods have been developed to reduce friction and wear. One of the most promising solutions is the introduction of surface texturing on a friction pair. The benefits of surface texturing and the effects of texturing parameters on tribological performance have been experimentally and theoretically investigated over the past two decades. Experimental investigations by means of friction tests were performed to study the influence of surface texturing on load-carrying capacity, friction forces and the friction coefficient [2–4]. Meanwhile, theoretical models were established to describe the mechanism behind surface texturing to improve the tribological performance of lubricated contacts [5–9]. In Kligerman and Etsion’s study [5], the pressure distribution in the uniform clearance between the annular surfaces is obtained from a solution of the Reynolds equation for compressible viscous gas in a laminar flow. Wang et al. [6] calculated the dimensionless load of a SiC thrust bearing based on the Reynolds equation and compared the results with the experimental results. Rahmani et al. [7] presented a method of integrating the Reynolds equation for partially textured slider bearings to achieve the optimum texturing parameters. In Meng and Khonsari’s study [9], the Stokes equations and the energy equation were solved for the microtextured parallel surfaces to gain insight into the role of the viscosity wedge on the pressure distribution and the load-carrying capacity.

From the experimental and theoretical studies, it was found that properly designed surface texture acts as micro-hydrodynamic bearings on the friction pair, which help to reduce friction and increase the load-carrying capacity. In addition, the surface texturing also provides extra space to reserve lubricant and entrap wear debris. Furthermore, parametric studies were conducted for various applications such as thrust bearings [10–12], journal bearings [13], engine cylinders [14–17] and mechanical seals [3,18]. It was concluded that there are optimal texturing parameters with which the friction pair exhibits optimal tribological performance.

As shown in this literature survey, most theoretical studies are based on the Reynolds equation. In some cases, textured surfaces under hydrodynamic lubrication can be accurately modeled by the Reynolds equation. However, the Reynolds equation is not accurate when inertial effects are important, for instance, when the Reynolds number is high, when the textures a have high aspect ratio, or when the texture depth exceeds 10% of the film thickness [19–21]. In these cases, the general Navier–Stokes (N-S) equations have to be adopted in order to account for the role of convective inertia in generating lift from surface textures.

Due to the development of more efficient algorithms and computational techniques in recent years, the mechanisms and effects of surface texturing on improving tribological performance could be numerically investigated using CFD based on the N-S equations.

Based on a two-dimensional CFD method, the tribological performance of surface with groove-shaped texture was studied. Sahlin et al. [22] successfully compared the lubrication characteristics of groove texture with arc-shaped cross section against that with spline-shaped cross section. The pressure distribution of the film and the fluid field status in grooves were investigated. Moreover, the effects of groove depth, groove width and Reynolds number on load-carrying capacity were studied. Brajdic-Mitidieri et al. [23] investigated the effect of groove-shaped texture on the lubrication characteristics of unparallel sliding surfaces. It was found that surface texturing not only reduces the friction coefficient significantly, but also improves the load-carrying capacity. Li and Chen [20] studied the lubrication characteristics of parallel surfaces with rectangle-shaped grooves. The results calculated using the two-dimensional CFD method were compared against those based on the Reynolds equations. It was found that when the groove depth is greater than ten percent of the film thickness, the method based on the Reynolds equations is no longer applicable. Shi and Ni [24] developed a two-dimensional CFD model to investigate the effects of groove texture on fully lubricated sliding with cavitation. The effects of cavitation pressure, sliding speed, sliding pitch angle and texture scale were discussed. Ramesh et al. [25] solved the N-S equations by using two-dimensional CFD and predicted the texture-induced lift. The results showed good correlation between the experiments and the CFD analysis. The above-described studies are all focused on groove-textured surfaces using a two-dimensional method.

It was reported that the tribological performance of a dimple-textured surface is better than that of groove-textured surface in terms of the friction coefficient [26]. A two-dimensional method is more suitable to simulate the hydrodynamic lubrication of a groove-textured surface, in which case the cross section of the surface is identical along the groove length. Due to the geometrical complexity of the micro-dimple array, however, the commonly used two-dimensional CFD technique is not able to simulate the lubrication behavior accurately. Therefore, the three-dimensional CFD method was proposed. Han et al. [27] studied the tribological characteristics of the micro-dimple array by using a three-dimensional CFD technique. The effects of micro-dimple size and the Reynolds number on film pressure, friction force as well as the friction coefficient were investigated and the optimum range for the micro-dimple depth was recommended.

Published papers regarding hydrodynamic lubrication of micro-dimple textured surfaces using three-dimensional CFD are very limited. Moreover, although some general guidelines for finding the optimal texturing parameters exist, the effects of texturing parameters on the tribological performance are highly dependent on contact and operating conditions [28]. Therefore, it is necessary to put more efforts into numerically investigating the hydrodynamic lubrication of micro-dimple textured surface.

In this study, the three-dimensional CFD method based on the N-S equations is proposed. The purpose of this study is to simulate the tribological behavior of a friction pair with a micro-dimple array under the condition of hydrodynamic lubrication. The pressure and velocity distribution are obtained using the finite volume method. Negative pressure is permitted and cavitation is not yet considered. The main mechanism for the improvement of the tribological performance by micro-dimple texturing is investigated and the optimum combination of texture density and aspect ratio for a given Reynolds number is discussed.

## Modeling

### Geometrical modeling

**Figure 1:**
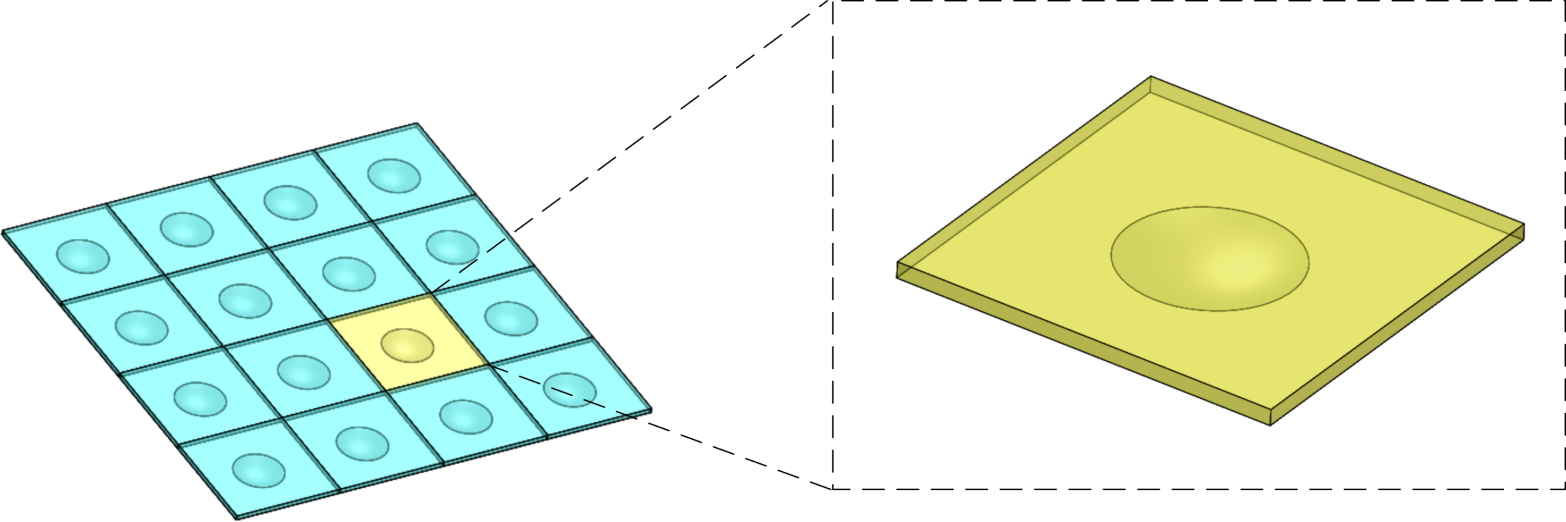
Three-dimensional geometric model of fluid in a micro-dimple array and a single micro-dimple unit.

In this study, the lubricant is filled between the upper flat surface and the lower surface in each compartment of the micro-dimple array. Because the micro-dimple array is composed of many identical micro-dimples which are linearly arranged with equal intervals, the fluids can also be divided into many identical units. Figure 1 shows the three-dimensional geometric model of the fluid in the micro-dimple array and details of a single micro-dimple unit.

**Figure 2:**
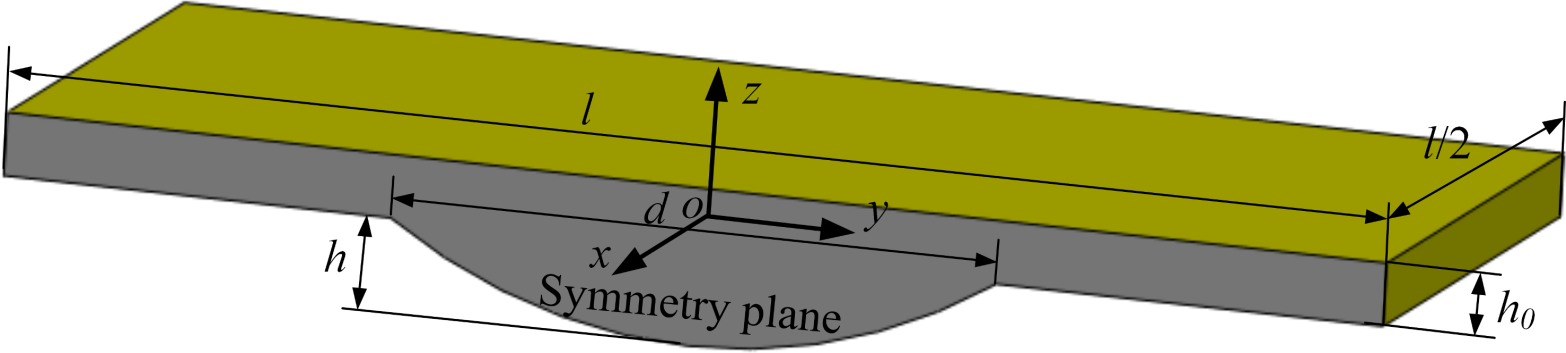
The computational domain.

As shown in Figure 2, due to the symmetrical characteristic of each micro-dimple unit, only half of the fluid in the micro-dimple unit is chosen as the computational domain. The coordinate system and geometrical parameters of the computational domain used in this study are also shown in Figure 2. The origin of the coordinate system is located on the revolution axis of the micro-dimple and is *h*_0_/2 away from the upper surface. The *x*, *y* and *z* axes are defined as the direction vertical to the symmetry plane, the flow direction and the direction of lubrication film thickness, respectively. *l* is the characteristic length of a micro-dimple unit, *h*_0_ is the thickness of lubricant film, which is identical to the gap between the friction pairs, *h* is the depth of the micro-dimple and *d* is the diameter of dimple.

### Mathematical modeling

#### Governing equations

In order to solve the hydrodynamic lubrication problem for a friction pair with micro-dimple texturing more accurately, the N-S equations, which are the equations of momentum conservation considering inertia terms, are used as the governing equations. In addition, the behavior of the lubricant in a micro-dimple array satisfies the law of mass conservation.

For the purpose of facilitating modeling and analysis, the following assumptions were made: 1) The body force is considered negligible (e.g., gravity or magnetic force); 2) No slip of lubricant is supposed to occur on the boundary, which means the velocity of lubricant close to the friction pair surface is identical with that of the friction pair surface; 3) The isothermal condition is considered and the lubricant is assumed to be an incompressible Newtonian fluid.

Based on these assumptions, the N-S equations and the continuation equation could be simplified. Furthermore, in order to reduce the number of independent variables, the dimensionless variables are defined as follows:

[1]
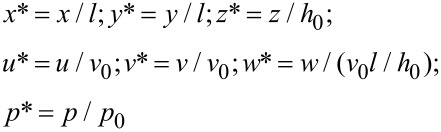


where *u*, *v* and *w* are the velocities of the fluid along the *x*, *y*, and *z* axes, respectively; *η* is the dynamic viscosity; *p* is pressure; *v*_0_ is the characteristic velocity of the lubricant and *p*_0_ is the characteristic pressure.

By substituting the above dimensionless variables into the simplified N-S equations [29], the dimensionless N-S equations can be expressed as the following. Along the direction of the *x*-axis:

[2]
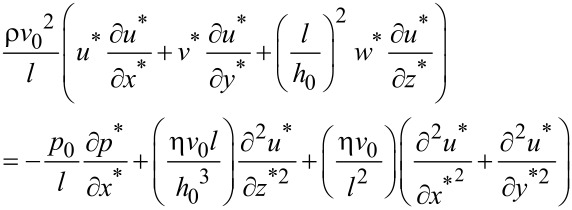


where ρ is density of the fluid. By letting *p*_0_ = ην_0 _*l*/*h*_0_^2^ and defining the Reynolds number as *Re* = ρν_0_*h*_0_/η, the above equation can be simplified as:

[3]
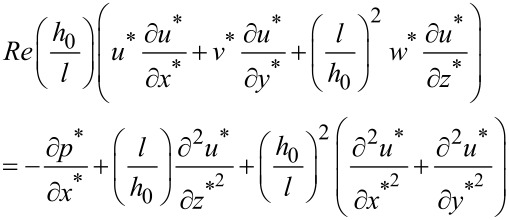


In a similar way, the equations along the *y* and *z*-axis could be obtained as follows:

[4]
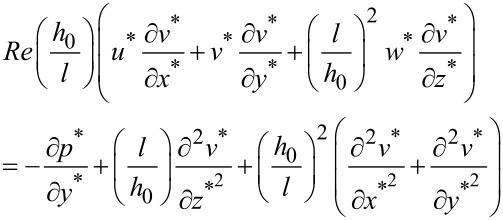


[5]
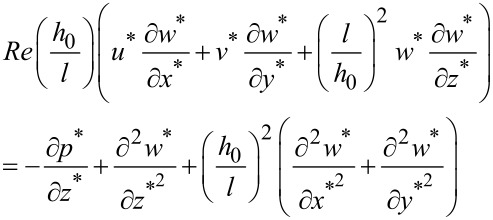


The dimensionless continuity equation could be expressed as:

[6]
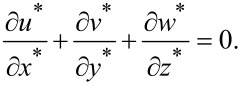


Equations 3–6 could be used to describe the behavior of the fluid in micro-dimple unit under the condition of hydrodynamic lubrication. Due to the complexity of the governing equations, however, it is difficult to obtain an analytical solution. Therefore, a numerical simulation is adopted in this study to solve the problem.

#### Characterization of tribological properties

The effect of the micro-dimple array on the tribological performance of a friction pair under the condition of hydrodynamic lubrication can be characterized by the following tribological characteristics: (1) dimensionless average film carrying force; (2) dimensionless average film shear force; (3) friction coefficient.

The film carrying force is calculated by integrating the pressure on the upper wall over the total calculation domain and can be expressed by

[7]
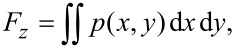


where *F_z_* is the film carrying force and *p*(*x*,*y*) is the pressure distribution function. The dimensionless form of the average film carrying force is obtained by

[8]
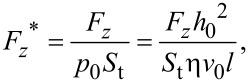


where *S*_t_ is the area of the upper wall of the calculation domain, that is, the area of the micro-dimple unit. A high dimensionless average film carrying force indicates a good load-carrying capacity of the friction pair.

In a similar way, as shown in Equation 9, the film shear force is calculated by integrating the shear stress along the *y*-axis on the upper wall over the total calculation domain:

[9]
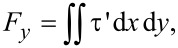


where *F_y_* is shear force and τ′ is the shear stress. The dimensionless form of the average film shear force can be obtained by

[10]
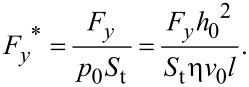


The friction coefficient is defined as the ratio of the dimensionless average film shear force to the dimensionless average film carrying force and can be expressed as

[11]
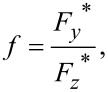


where *f* represents the friction coefficient. A low friction coefficient means small shear force with large carrying force, which indicates good behavior of the comprehensive tribological performance under the condition of hydrodynamic lubrication.

### Numerical simulation

#### Meshing

**Figure 3:**
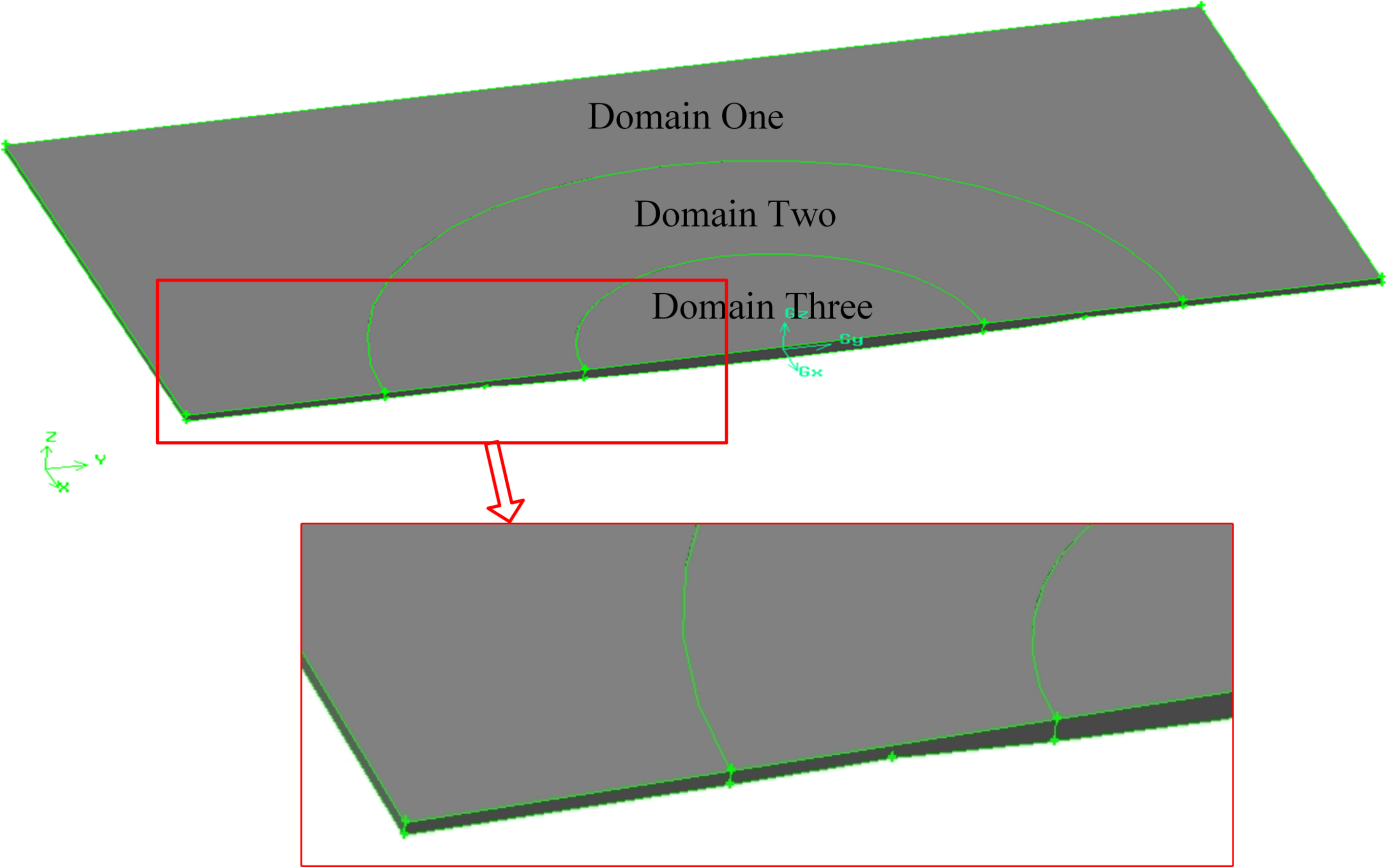
The division of the fluid domain in the micro-dimple unit.

The three-dimensional model of the computational domain was meshed by using GAMBIT software. In order to obtain a high-quality mesh, the model was divided into three domains (see Figure 3). The micro-dimple was located in domain two and domain three.

In order to guarantee the accuracy of calculation and the rate of convergence, the meshing operation follows several criteria: (1) the use of structured grids, as far as possible; (2) the ratio of the length of the longest element edge to the length of the shortest element edge should be controlled below 5:1; (3) the skewness should be controlled to be no more than 0.9; (4) because the entry, exit and interior of the dimple are the areas of most concern, the mesh density of the model increases gradually from domain one to domain three; (5) the dimensional difference between neighboring elements should be controlled to be as small as possible.

The meshing is challenging in particular for shallow dimples and for a small film thickness. A smaller dimple depth requires very small grid volumes in order to have enough volume in the vertical direction. When combined with the volume aspect ratio limits, a large number of volumes are required to fill the horizontal extent of the dimple. Since the mesh size has a great effect on the accuracy and efficiency of the numerical simulation, the grid independence analysis was carried out to develop the proper meshing strategy. The variation of the mesh size may affect the film pressure distribution and therefore the load-carrying capacity. Thus, the dimensionless average film carrying force, *F*_z_*, was considered as the main indicator of grid independence. The mesh sizes were chosen to suit the geometry of each case so that further mesh refinement did not change the dimensionless average film carrying force by more than 0.01%.

**Figure 4:**
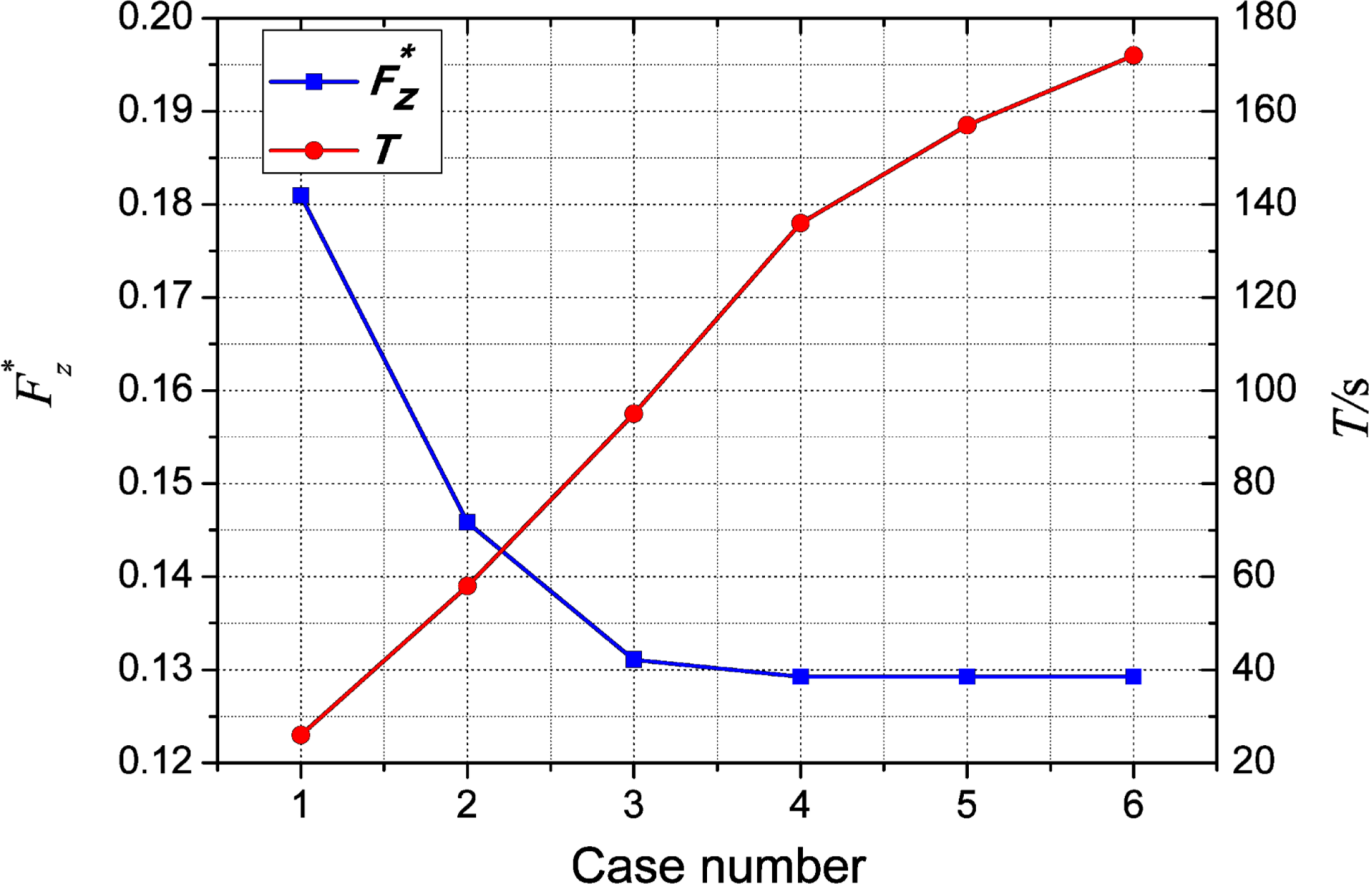
Variations of the dimensionless average film carrying force and the computing time.

Based on the typical dimple unit geometry characterized by *l* = 2400 µm, *d* = 1200 μm, *h* = 20 μm and *h*_0_ = 20 μm, the grid independence analysis was performed as follows. Six sets of mesh sizes were selected for calculation (see Table 1). The variations of the dimensionless average film carrying force, *F*_z_*, and the computing time, *T*, of the simulation with different average mesh sizes are shown in Figure 4.

**Table 1:** Mesh sizes for the grid independence analysis.

Case number	Average mesh size (μm)		
	
	Domain one	Domain two	Domain three

1	12	10	8
2	10	8	6
3	8	6	4
4	6	5	4
5	6	4	3
6	5	4	3

**Figure 5:**
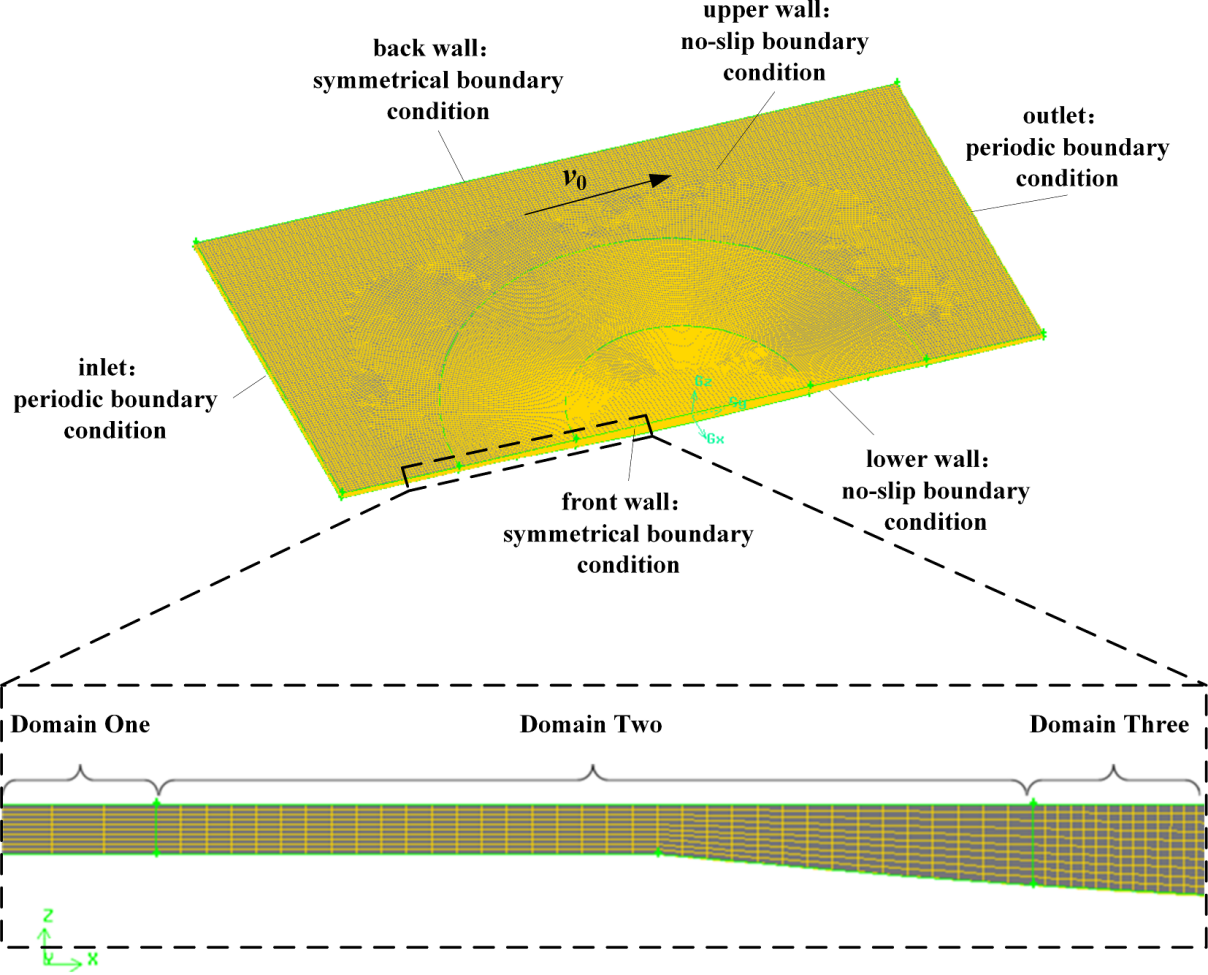
The typical meshed model of a micro-dimple unit.

It is noted from Table 1 that the average mesh size decreases gradually from case 1 to case 6. As shown in Figure 4, from case 1 to case 6, *F*_z_* decreases and tends to be stable while *T* increases constantly. From case 4 to case 6, the value of *F*_z_* converged within a satisfactory tolerance of about 0.01% to a constant value. Meanwhile, the value of *T* for case 4 is in the acceptable range. Therefore, the average mesh sizes for case 4 were chosen for meshing this model. Figure 5 shows the meshed model of the micro-dimple unit with a total grid number of 416,670.

#### Boundary conditions

As shown in Figure 5, the upper and lower walls of the simulation model are set as no-slip boundaries. The upper wall moves along the positive direction of the *y*-axis with a velocity of *v*_0_, while the lower wall is assumed stationary. The inlet and outlet of the model along the *y*-axis are set as periodic boundary conditions. That is, the values of the variables of the inlet are the same as those of the outlet. The two walls along the *x*-axis are set as symmetrical boundary conditions.

#### Calculation

The model is calculated by utilizing commercial CFD software, and the FLUENT and SIMPLE algorithms were adopted for solving. The key parameters used in the CFD simulation are listed in Table 2.

**Table 2:** Parameters of the CFD simulation.

Texture density, ρ*_t_*	5%, 13%, 20%, 50%
Aspect ratio, λ	0.017, 0.033, 0.05, 0.075, 0.1, 0.125, 0.2
Reynolds number, *Re*	5, 50, 250

In Table 2, the texture density is defined as the ratio of the micro-dimple area to the friction pair area. Because the micro-dimple array could be divided into many identical micro-dimple units, the texture density can be expressed as

[12]
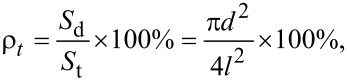


where *S*_d_ is the area of a single micro-dimple and *S*_t_ is the area of a single micro-dimple unit. The aspect ratio, λ, is defined as the ratio of the micro-dimple depth to the micro-dimple diameter.

## Results and Discussion

### Film pressure distribution of the micro-dimple unit

**Figure 6:**
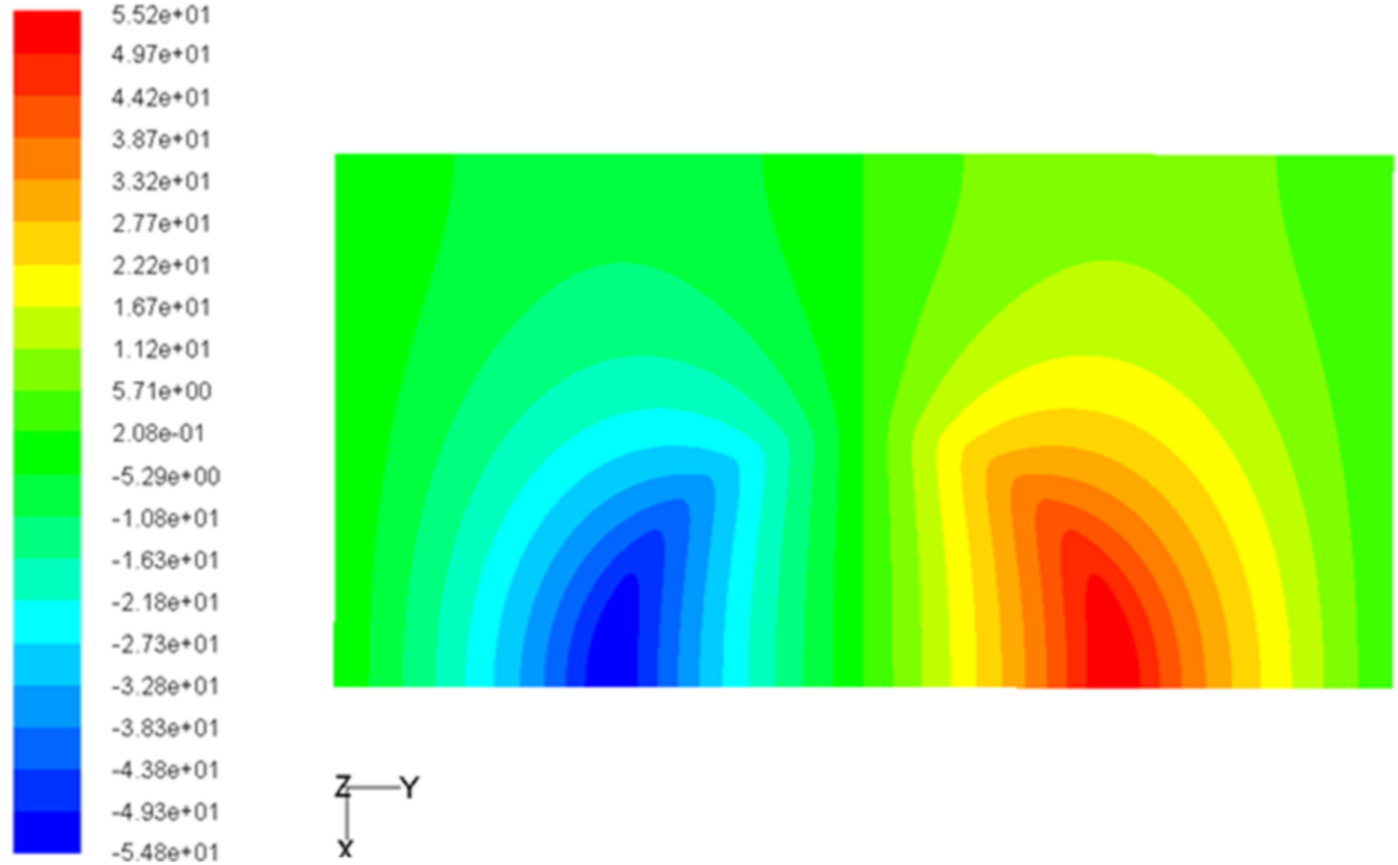
Pressure distribution on the upper wall of the lubricant in the micro-dimple unit.

**Figure 7:**
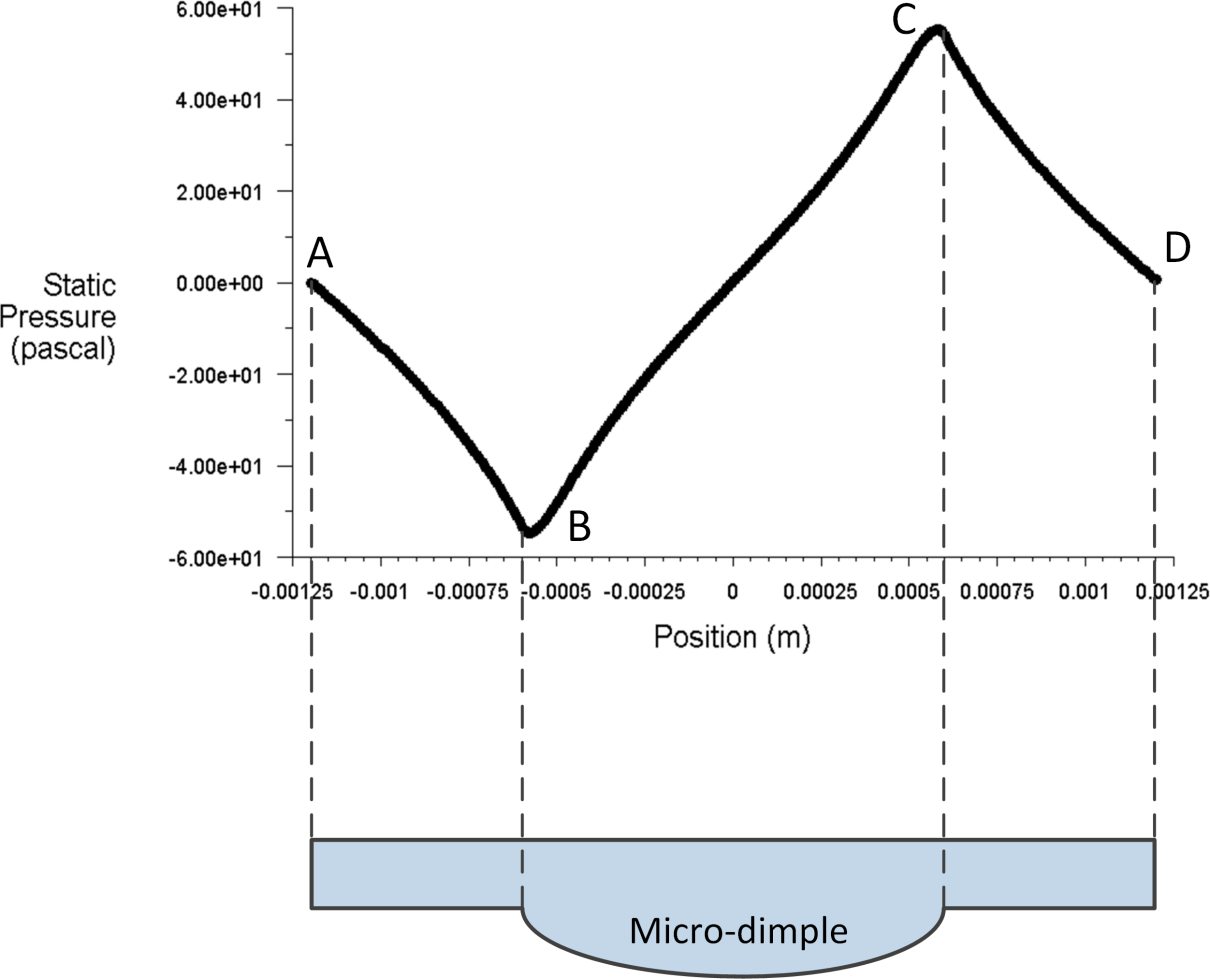
Pressure distribution of the cross-sectional area of the lubricant in a micro-dimple unit.

The typical pressure distribution on the upper wall of the lubricant in the micro-dimple unit is shown in Figure 6. Moreover, Figure 7 shows the pressure distribution on the middle section of the micro-dimple unit. From Figure 6 and Figure 7, it is can be seen that when the lubricant flows into the micro-dimple unit from point A to point D, the pressure decreases gradually and reaches a minimum at the entry of the micro-dimple (point B). Then the pressure starts to increase and reaches a maximum at the exit of the micro-dimple (point C). From point C to point D, the pressure decreases to a value at point D, which is identical as that at point A.

Positive hydrodynamic pressure is generated in the convergent part of the gap while negative hydrodynamic pressure is generated in the divergent part. With suitable parameters (e.g., shape and size of the micro-dimple unit, film thickness and velocity of fluid), the magnitude of the positive pressure could be greater than that of the negative pressure, which makes the total integration of the pressure in the micro-dimple unit become positive. This phenomenon is called the wedging effect of a micro-dimple. In this case, the hydrodynamic pressure in the micro-dimple unit offers extra carrying force, which helps increase the load-carrying capacity of the friction pair.

### The effect of Reynolds number on the dimensionless pressure distribution

**Figure 8:**
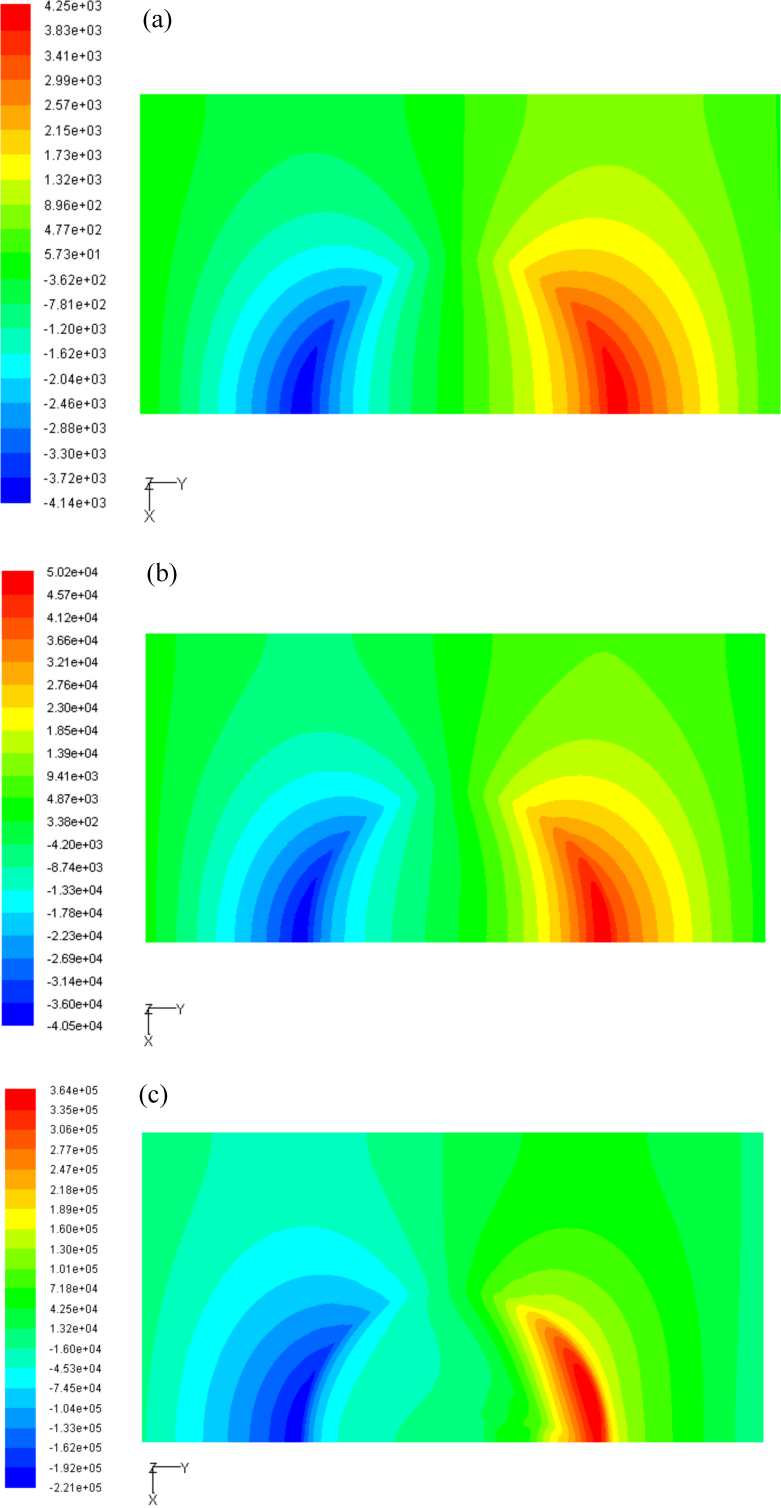
The pressure distribution on the upper wall for (a) *Re* = 5, (b) *Re* = 50 and (c) *Re* = 250.

**Figure 9:**
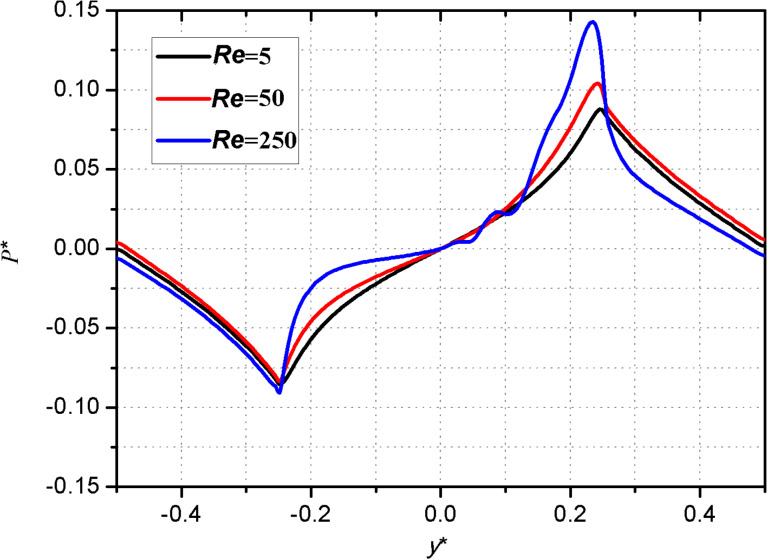
Effect of Reynolds number on the pressure distribution on the middle section of the micro-dimple unit.

In this study, the Reynolds number is changed by modifying the moving velocity of the upper wall. The three-dimensional pressure distribution on the upper wall under different Reynolds numbers is shown in Figure 8. Figure 9 presents the two-dimensional pressure distribution on the middle section of the micro-dimple unit. From Figure 8 and Figure 9, it can be seen that the Reynolds number affects the dimensionless pressure distribution significantly. The magnitude of the positive and negative pressure increases with Reynolds number. The increase rate of the positive pressure, however, is higher than that of the negative pressure. This result indicates that a large Reynolds number leads to a high hydrodynamic pressure.

### Effect of texture density and aspect ratio on the dimensionless average film carrying force

**Figure 10:**
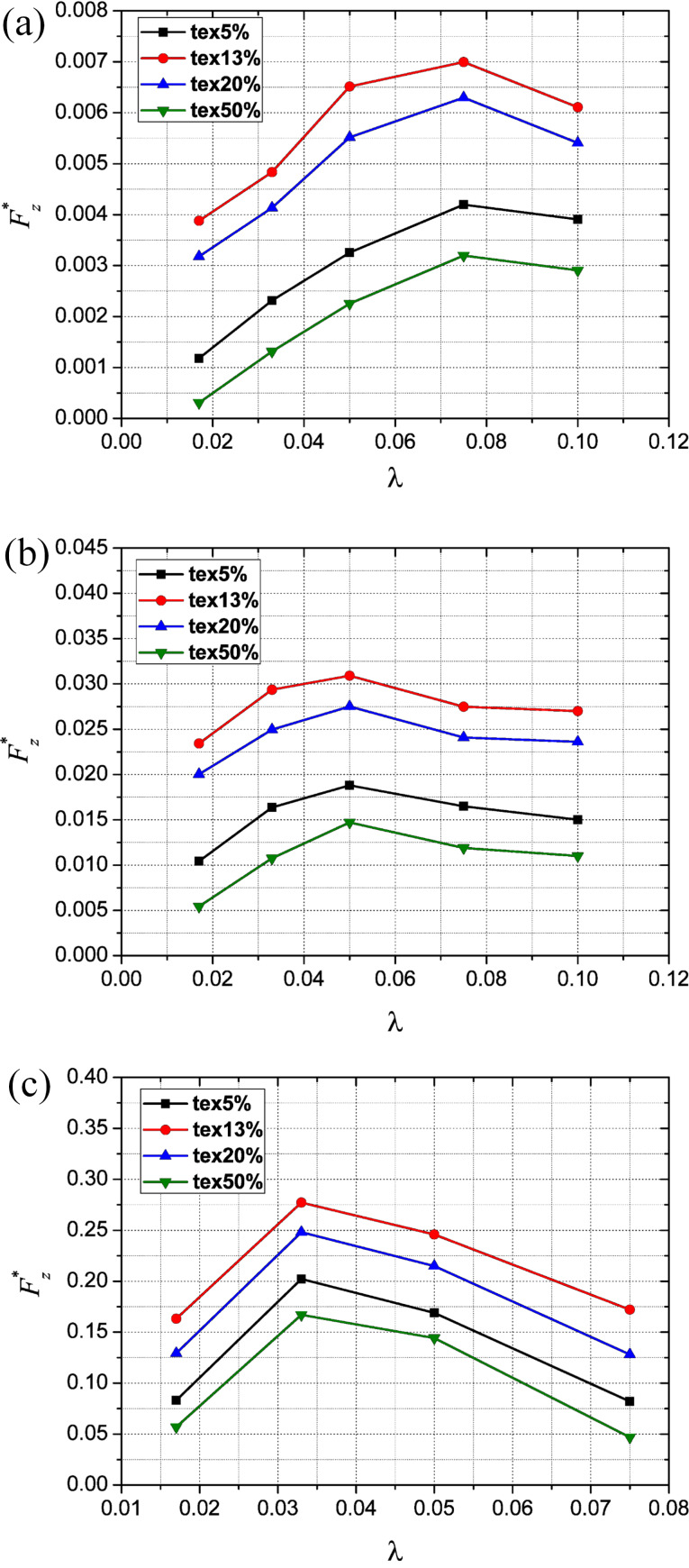
Effect of texture density and aspect ratio on the dimensionless average film carrying force for (a) *Re* = 5, (b) *Re* = 50 and (c) *Re* = 250.

Figure 10a–c shows the effect of texture density and aspect ratio on the dimensionless average film carrying force with Reynolds numbers of 5, 50, and 250, respectively (the texture density of 5%, 13%, 20% and 50% are abbreviated as tex5%, tex13%, tex20% and tex50%, respectively).

It is found that for a given Reynolds number and aspect ratio, the dimensionless average film carrying force at a texture density of 13% is higher than that at a texture density of 5%, 20% and 50%. This indicates that there exists an optimum texture density which leads to a maximum carrying force. Meanwhile, it can be seen that with a given Reynolds number and texture density, there also exists an optimum aspect ratio at which the film carrying force reaches a maximum. However, the optimum aspect ratio is not constant but changes with the Reynolds number. The existence of an optimum texture density and optimum aspect ratio is also observed and proved using experimental and analytical methods in previously published investigations [3,6,30].

The reason for the existence of an optimum aspect ratio could be explained as follows. By assuming that the diameter of the micro-dimple is constant, a large aspect ratio means a large micro-dimple depth. The inertia force of the lubricant in the micro-dimple unit increases with the depth, which can enhance the wedging effect that is helpful for improving the load-carrying capacity. However, when the depth increases to a certain value, recirculation starts to occur at the bottom of the micro-dimple due to the interaction between the fluid and the wall of micro-dimple. In terms of energy conversion, part of the energy transferred from the moving film upper wall to the fluid is dissipated by being converted to the kinetic energy of recirculation. Therefore, the load-carrying capacity is reduced. This phenomenon could be called the recirculation effect. Due to the joint result of the wedging effect and the recirculation effect, there is an optimum value of the aspect ratio which leads to the best load-carrying capacity of the micro-dimple unit.

**Figure 11:**
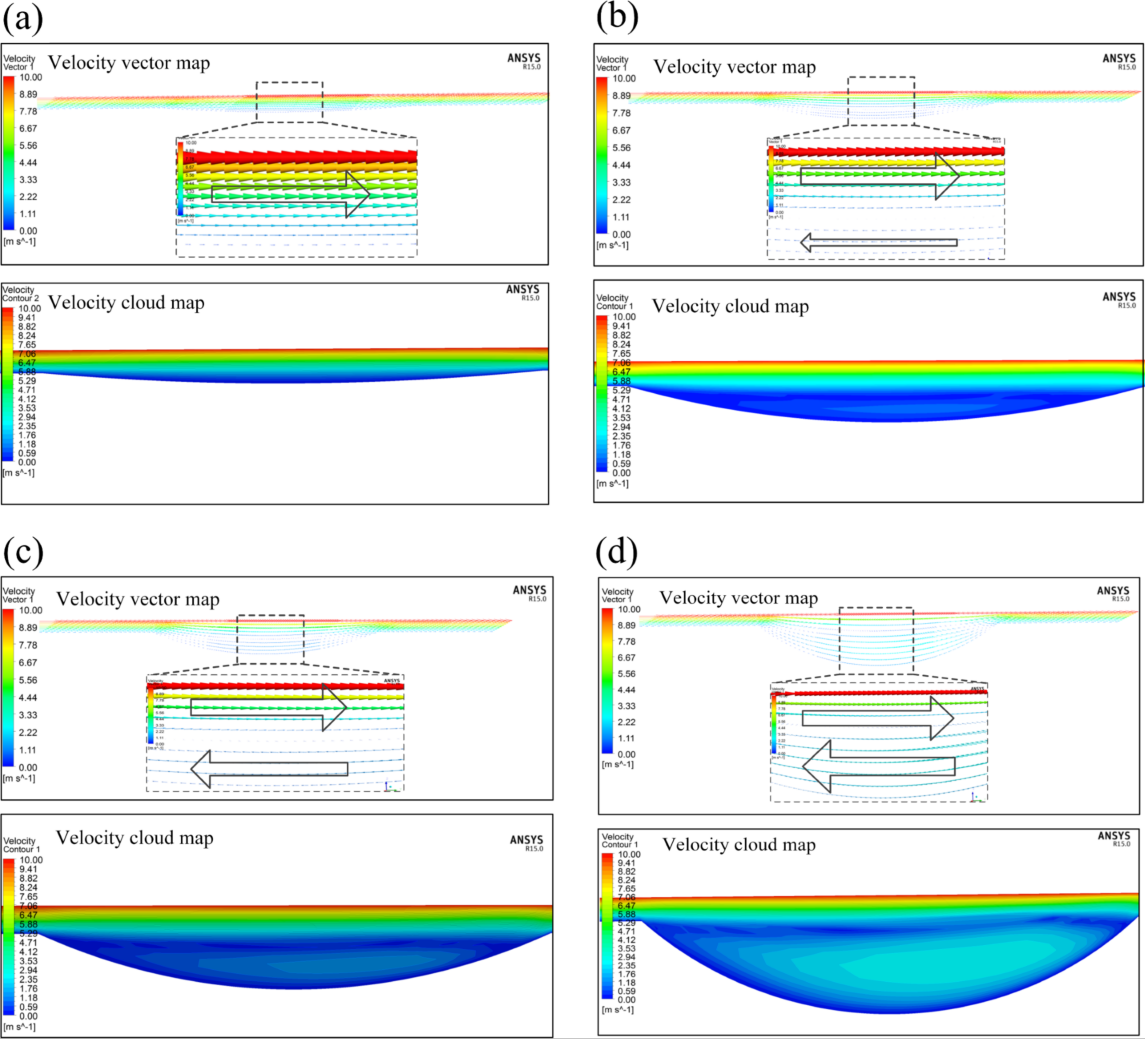
Variation of the fluid velocity in the micro-dimple unit with (a) λ = 0.025, (b) λ = 0.075, (c) λ = 0.1 and (d) λ = 0.2 (texture density: 20%).

To further investigate the recirculation effect, the velocity streamlines on the middle section of the micro-dimple unit with different aspect ratios are compared in Figure 11. The arrow direction indicates the direction of velocity and the arrow size stands for the velocity magnitude.

With a small aspect ratio, the direction of the fluid velocity at the bottom of the micro-dimple is identical with that on the upper wall (see Figure 11a). When the aspect ratio is increased, it is found that the direction of fluid flow at the bottom of the micro-dimple is reversed to that of the upper wall, which implies the generation of a recirculation zone in the micro-dimple (see Figure 11b). With a further increase of the aspect ratio, the range of the recirculation zone increases. This means that more energy is converted to kinetic energy of recirculation, resulting in a more adverse effect on the load-carrying capacity of the micro-dimple (see Figure 11c,d).

### Effect of texture density and aspect ratio on the dimensionless average film shear force

**Figure 12:**
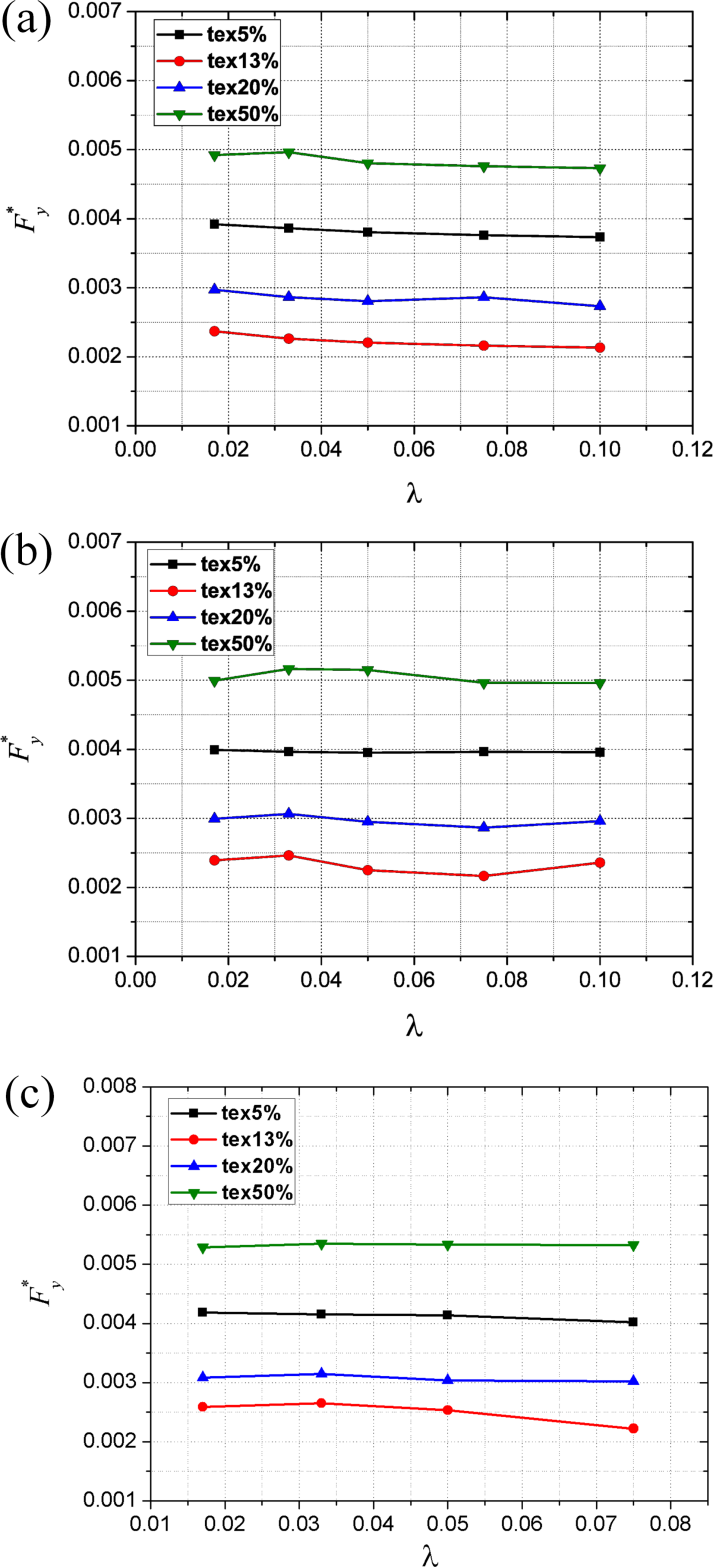
Effect of texture density and aspect ratio on the dimensionless average film shear force for (a) *Re* = 5, (b) *Re* = 50 and (c) *Re* = 250.

Figure 12a–c shows the effects of the texture density and aspect ratio on the dimensionless average film shear force with Reynolds numbers of 5, 50 and 250, respectively.

At a given Reynolds number and aspect ratio, with the increase of texture density, the dimensionless average film shear force decreases first and then starts to increase after reaching a minimum. It is noted that the variation trend of the dimensionless average shear force is opposite to that of the dimensionless average film carrying force. In addition, with a given Reynolds number and texture density, the variation of aspect ratio has very limited effect on dimensionless average shear force.

### Effect of texture density and aspect ratio on friction coefficient

**Figure 13:**
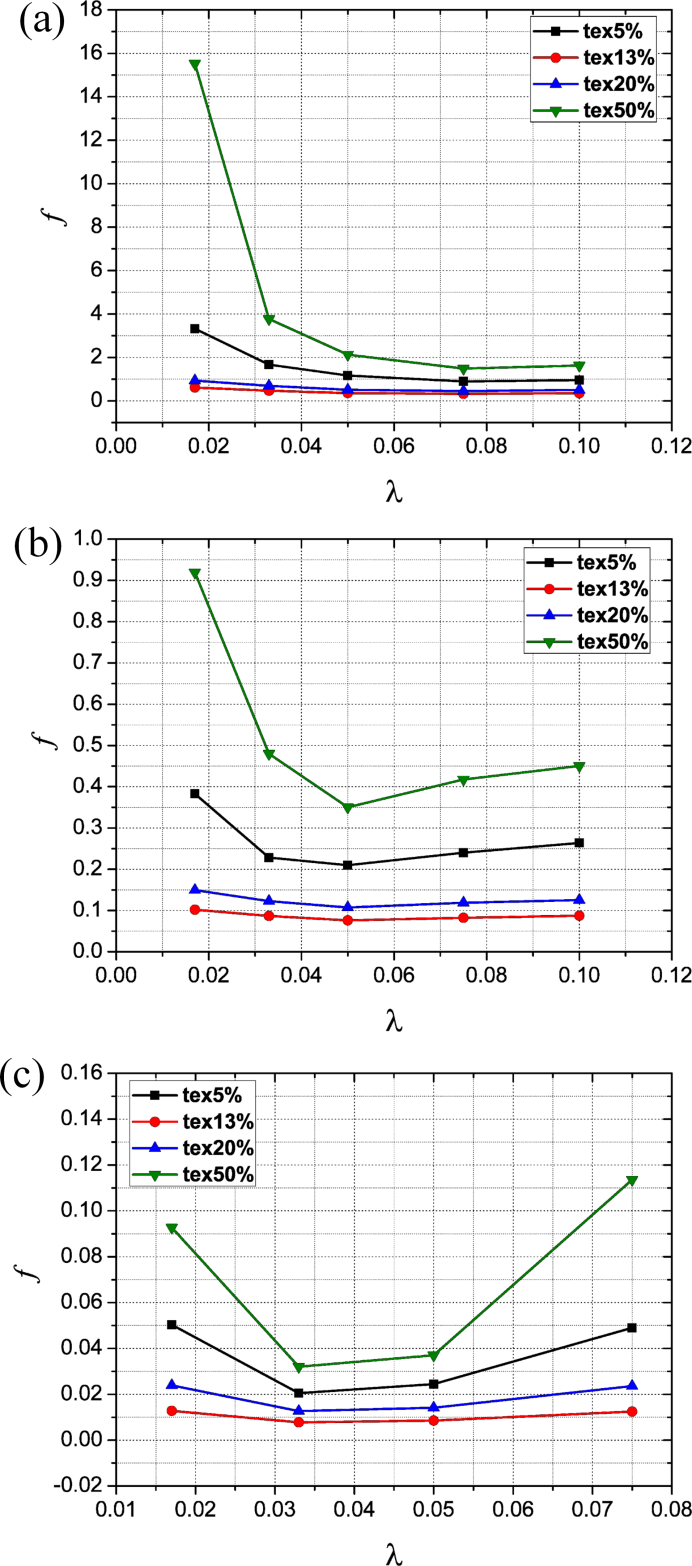
Effect of texture density and aspect ratio on the friction coefficient for (a) *Re* = 5, (b) *Re* = 50 and (c) *Re* = 250.

The effect of texture density and aspect ratio on the friction coefficient with Reynolds numbers of 5, 50 and 250 is shown in Figure 13a–c, respectively. Due to the insignificant impact of texture density and aspect ratio on the dimensionless average film shear force, it is found that the variation trend of the friction coefficient is reverse to that of the dimensionless average film carrying force. There also exists an optimum texture density and optimum aspect ratio which leads to the best tribological performance. Within the range of parameters investigated in this study, the optimum texture density was found to be 13% while the optimum aspect ratio varies with the Reynolds number.

The above conclusion is in good agreement with other experimental investigations [31] in which the effect of texture density on the friction coefficient was studied. It was found that the lowest friction coefficient was achieved with a texture density of 10.4% when comparing against those with a texture density of 2.6%, 15.5% and 22.9%.

### Effect of Reynolds number on the tribological characteristics of the friction pair

**Figure 14:**
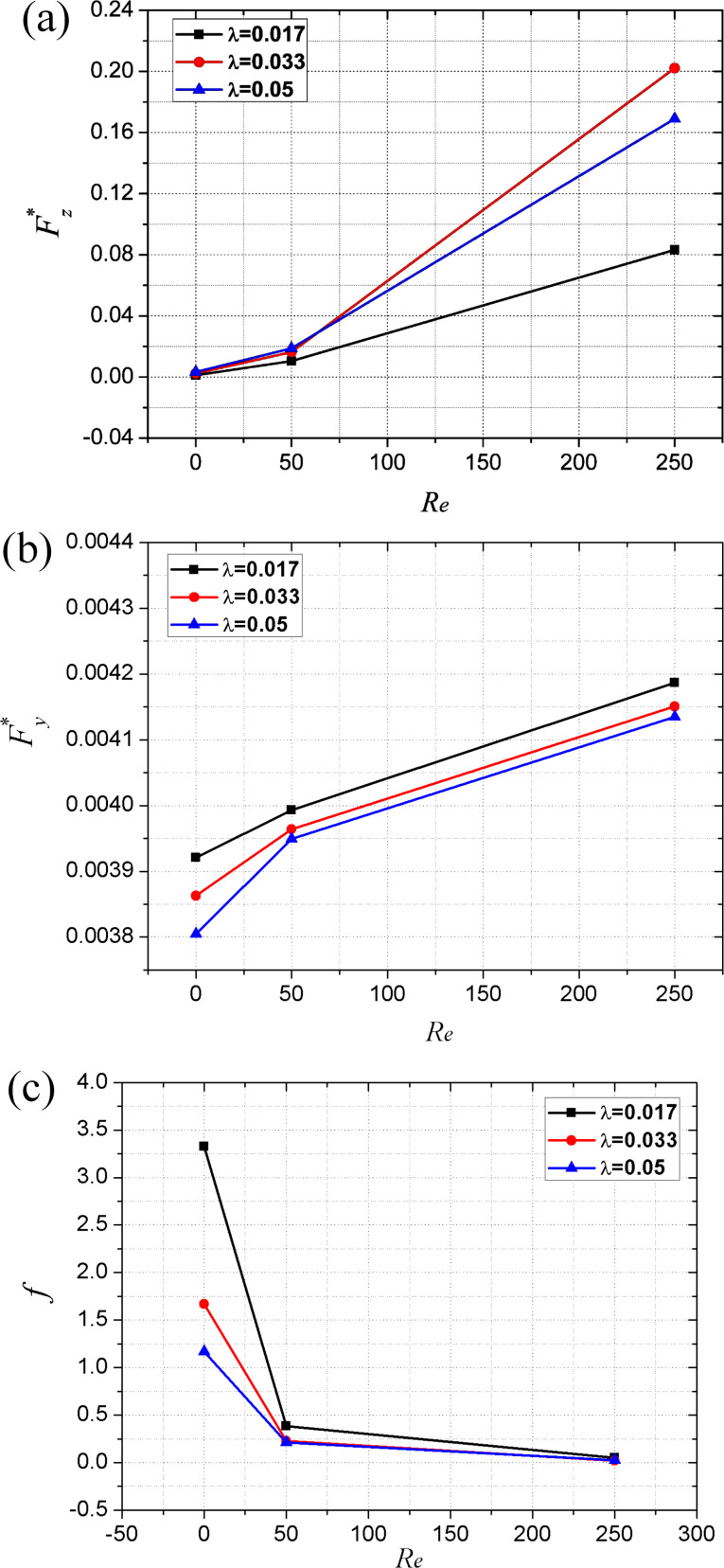
Effect of Reynolds number on (a) the dimensionless average film carrying force, (b) the dimensionless average film shear force and (c) the friction coefficient.

Figure 14a–c illustrates the trend variation of the tribological characteristics with Reynolds number for the cases with a texture density of 13% and aspect ratio of 0.017, 0.033 and 0.05.

It can be seen from Figure 14a that the dimensionless average film carrying force obviously increases with Reynolds number. In addition, the increasing rate of the carrying force increases with the value of the Reynolds number. As shown in Figure 8a–c, with an increase in the Reynolds number, the maximum magnitude, as well as the distributed area of positive pressure, increases more significantly than that of the negative pressure. As a result, the total integration of the pressure over the area of film upper wall (which stands for the film carrying force) increases with the Reynolds number.

Although the dimensionless average film shear force increases with Reynolds number as well, the increase rate is very small as compared to that of the dimensionless average film carrying force (see Figure 14b). When the Reynolds number increases from 5 to 50, and from 50 to 250, the dimensionless average carrying force increases by 606% and 1135%, respectively. However, the dimensionless average film shear force increases by only 3% and 5%, respectively. These conclusions about the effect of the Reynolds number on the carrying force and shear force are consistent with those in another study [27]. As a result, the variation of the friction coefficient with Reynolds number is reverse to that of the dimensionless average film carrying force (see Figure 14c).

### Effect of Reynolds number on optimum texture density and optimum aspect ratio

**Figure 15:**
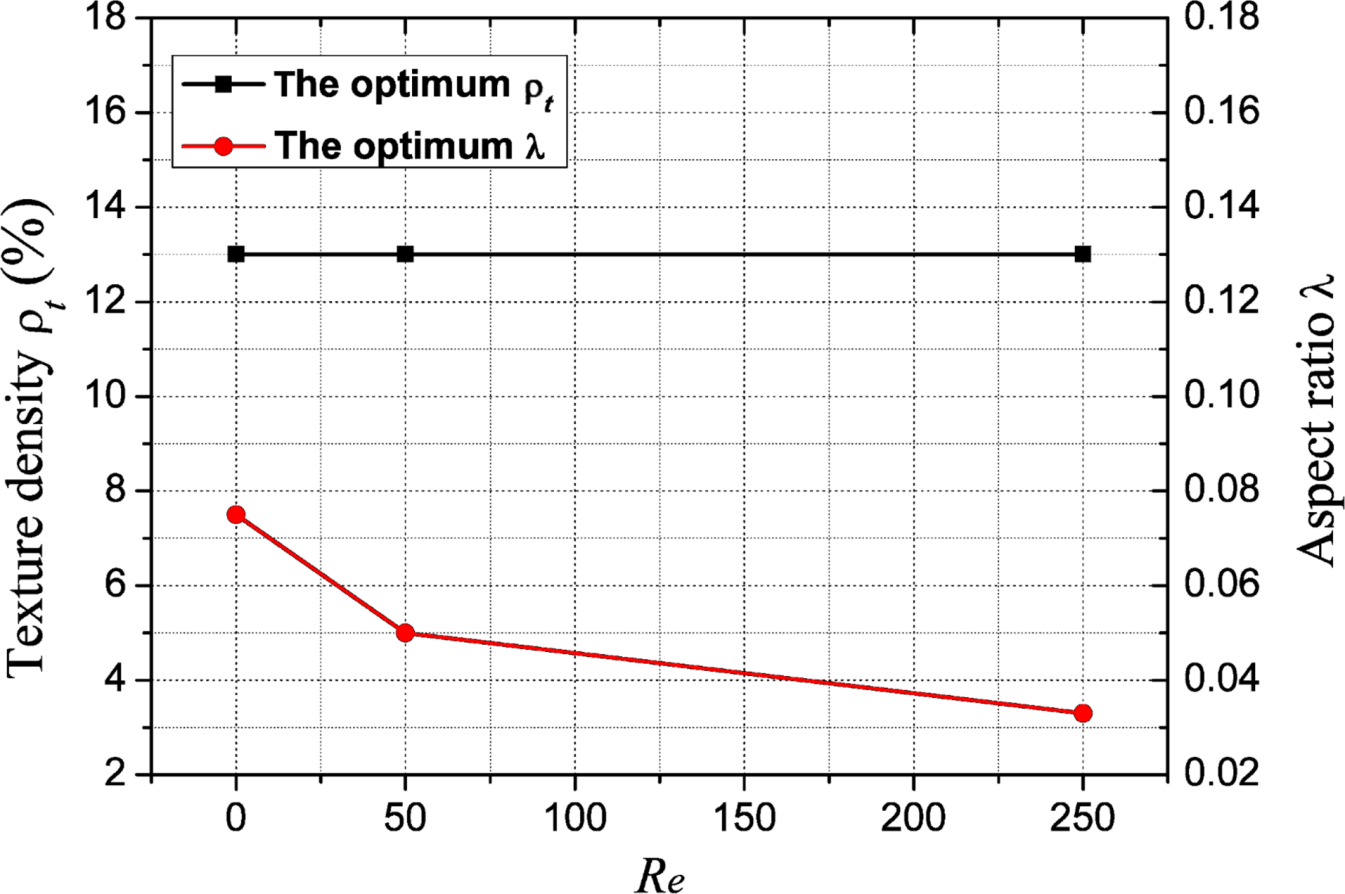
Effect of Reynolds number on the optimum texture density and optimum aspect ratio.

Figure 15 shows the variation of the optimum texture density and the optimum aspect ratio with Reynolds number. It is found that, within the parameter range of this study, the optimum texture density is independent of the Reynolds number and the micro-dimple array, where a texture density of 13% is found to exhibit the optimum hydrodynamic lubrication performance in all cases. The optimum dimple aspect ratio decreases with increased Reynolds number. For a given industrial application, the operating condition could be characterized by a corresponding Reynolds number. Therefore, for a given Reynolds number, the preferred dimensional parameters of micro-dimple texturing could be obtained according to the combination of optimum texture density and optimum aspect ratio, which leads to the best tribological performance.

## Conclusion

By utilizing a three-dimensional CFD simulation method, the tribological performance of a micro-dimple array under the condition of hydrodynamic lubrication was investigated. This study focused on the influence of the micro-dimple array parameters and fluid properties on the film pressure distribution and tribological characteristics. The main conclusions are as follows.

With proper dimensional parameters, such as texture density and aspect ratio, the total integration of the film pressure could be positive. As a result, the micro-dimple texture could provide extra carrying force, which is helpful for improving the tribological performance of a friction pair. The wedging effect of the micro-dimple texturing is beneficial for the load-carrying capacity, while the recirculation effect is disadvantage in this sense. The main mechanism for the improvement of the tribological performance is the comprehensive results of these two effects. For a certain Reynolds number, there exists the combination of an optimum texture density and optimum aspect ratio with which the maximum hydrodynamic lubrication coefficient could be obtained. In this case, the optimum tribological performance under the condition of hydrodynamic lubrication is achieved. And finally, it was concluded that within the parameters used in this study, when the Reynolds number is increased, the optimum aspect ratio decreases while the optimum texture density does not change.

The conclusions drawn in this study could be the basis for further numerical studies in specific applications such as thrust bearings, engine cylinders and mechanical seals. Our future work will focus on implementing more complex boundary conditions and thermal effects for specific applications.
